# Calibration of Integrated Low-Cost Environmental Sensors for Urban Air Temperature Based on Machine Learning

**DOI:** 10.3390/s25113398

**Published:** 2025-05-28

**Authors:** Fang Nan, Chao Zeng, Huanfeng Shen, Liupeng Lin

**Affiliations:** 1 School of Resource and Environmental Sciences, Wuhan University, Wuhan 430079, China; nf2020@whu.edu.cn (F.N.); shenhf@whu.edu.cn (H.S.); linliupeng@whu.edu.cn (L.L.); 2 Collaborative Innovation Center of Geospatial Technology, Wuhan 430079, China; 3 Hubei Luojia Laboratory, Wuhan University, Wuhan 430079, China; 4 Key Laboratory of Geographic Information System, Ministry of Education, Wuhan University, Wuhan 430079, China; 5 Key Laboratory of Digital Cartography and Land Information Application, Ministry of Natural Resources, Wuhan University, Wuhan 430079, China

**Keywords:** low-cost sensors, air temperature, calibration, machine learning

## Abstract

Monitoring urban microenvironments using low-cost sensors effectively addresses the spatiotemporal limitations of conventional monitoring networks. However, their widespread adoption is hindered by concerns regarding data quality. Calibrating these sensors is crucial for enabling their large-scale deployment and increasing confidence among researchers and users. This study focuses on an internet of things (IoT) application in Wuhan, China, aiming to enhance the quality of long-term hourly air temperature data collected by low-cost sensors through on-site calibration. Multiple linear regression (MLR) and light gradient boosting machine (LightGBM) algorithms were employed for calibration, with leave-one-out cross-validation (LOOCV) being used for model evaluation. Factors, such as multiple scenarios, spatial distances, and seasonal variations, were also examined for their influence on long-term data calibration. The experimental findings revealed that the LightGBM method consistently outperformed MLR. Calibration using this approach markedly improved the sensor data quality, with the R-squared (R^2^) value of the sensor with the poorest raw data increasing from 0.416 to 0.957, its mean absolute error (MAE) decreasing from 6.255 to 1.680, and its root mean square error (RMSE) being reduced from 7.881 to 2.148. This study demonstrates the application potential of using LightGBM as an advanced machine learning (ML) method in innovative low-cost sensors, thereby providing a method of obtaining high-quality and real-time information for urban environmental and public health research.

## 1. Introduction

The growing prevalence of climate change and extreme weather events has heightened the importance of meteorological monitoring [1,2]. Meteorological monitoring is essential for daily weather forecasting, providing disaster warnings, and supporting various activities that are critical to production and daily life. It provides fundamental data for characterizing and analyzing local climate patterns, with temperature being a particularly significant meteorological element.

Urbanization, a defining trend of the 21st century, is projected to double the urban population by 2050 [3]. While cities occupy only 2% of the Earth’s surface, urban residents are responsible for the majority of energy consumption, contributing to rising urban temperatures and the urban heat island effect [4,5]. This phenomenon poses significant challenges to achieving sustainable urban development [6,7]. Numerous studies on thermal comfort have emphasized the critical role of maintaining appropriate temperature and humidity levels for human health in both indoor and outdoor settings [8,9,10].

The increasing focus on urban temperature and outdoor thermal comfort has led to extensive research on the influence of urban geometry, the greening effect, and other factors in mitigating urban heat [8,11,12,13,14]. Despite the importance of accurate meteorological data, the sparse distribution of weather stations in many regions limits the availability of dense ground-level observations [15]. This gap often results in inconsistencies in localized temperature data, complicating urban heat island mitigation strategies [16]. Additionally, localized weather events that affect smaller areas present significant challenges to urban management [17]. In summary, conducting refined environmental monitoring at a granular scale within urban areas is critically important.

The advent of low-cost sensor technology in recent years has revolutionized the environmental monitoring landscape [18,19]. While standard monitoring networks established by official agencies provide highly accurate data, their instruments are expensive, complex, and require professional operation, regular maintenance, and strict environmental conditions [20,21,22]. These factors often result in a sparse geographical distribution of monitoring stations. Additionally, data from official agencies are often subjected to restricted access and significant time delays.

In contrast, low-cost sensors are approximately three-orders-of-magnitude less expensive than standard reference instruments [18], which enables their deployment across larger areas at higher densities. Given the substantial variability in urban microenvironments due to complex human activities, the economical and compact nature of low-cost sensors makes them an appealing choice for experiments and applications that require dense spatial mapping [23,24,25,26]. Studies utilizing low-cost sensors to examine the spatial variability of urban air quality [27,28] have demonstrated their efficacy in capturing environmental changes at fine spatial scales.

Low-cost sensors offer significant potential to address the spatial and temporal limitations of standard monitoring networks [29,30]. Deploying high-density, low-cost sensors in localized areas presents many benefits, but concerns about data quality hinder their widespread adoption [31,32,33]. Public and community interest in low-cost sensors is growing, yet the absence of reference instruments for comparison during their application [34] often results in unreliable data. These sensors are highly sensitive to environmental changes [34,35,36,37,38], and laboratory calibration alone fails to resolve these issues effectively [39]. Overall, improving the quality of the data collected by low-cost sensors through calibration is critical to enhancing user confidence and facilitating their large-scale application. Therefore, ML approaches have emerged as a leading approach in large-scale applications [19,40,41,42]. However, factors, such as the experiment duration, which is often overlooked, can affect calibration performance. Additionally, small-scale or unevenly distributed studies limit the generalization of calibration methods. Most studies on low-cost sensors focus on air quality [36,43,44,45], with relatively few studies leveraging urban meteorological data from low-cost sensor monitoring networks.

In past studies on the calibration and measurement of air temperature sensors, Liu et al. [46] proposed a calibration scheme and established a data calibration model using a backpropagation neural network, but the data only included 10 cloudy days in June and July, and only air temperature data was used as the input and output. Another study used typical liquid thermostats and climate chambers for measurements to determine the characteristics of a calibration system, but the cost of high-precision experimental equipment could not be ignored [47]. Sun et al. [48] developed a solar radiation lookup table, but the method relied on solar radiation data sensed by high-cost pyranometers and was only used to process 14 days’ worth of data. Yamamoto et al. [1] used an artificial neural network to balance the effects of multiple environmental factors on their measurements, but only in three different locations. Tang et al. [49] tried the LightGBM method to correct the numerical prediction results of their temperature prediction method temperature, but the study did not take into account the application advantages of low-cost sensors. Cao et al. [50] proposed a method for establishing a temperature-prediction model based on time series analysis, but it was mainly aimed at modern agricultural greenhouse systems. A previous case study of low-cost internet of things (IoT) environmental sensors in Wuhan, China, evaluated the accuracy of daily average air temperature data and tested the feasibility of a wireless integrated sensor network application [51]. However, detailed statistics and the calibration of hourly monitoring data remain underexplored, highlighting a gap in the application of low-cost sensors for urban meteorological monitoring.

In this study, we developed a calibration method for calibrating low-cost sensors for the long-term measurement of temperature, considering different locations and multiple environmental factors. First, the necessity of calibrating low-cost sensors is highlighted by comparing raw air temperature data from low-cost sensors with data from standard meteorological stations. Next, we calibrated a large dataset of hourly air temperature data from long-term field environments using MLR and LightGBM methods and compared the results with the original data to evaluate the obtained improvement. In addition, the calibration model’s migration capability was evaluated across different surface types and spatial locations. The findings indicate that the proposed calibration method significantly enhances the data quality of low-cost sensors and remains stable across diverse scenes and spatial locations.

## 2. Materials and Methods

### 2.1. Data Acquisition

**Figure 1:**
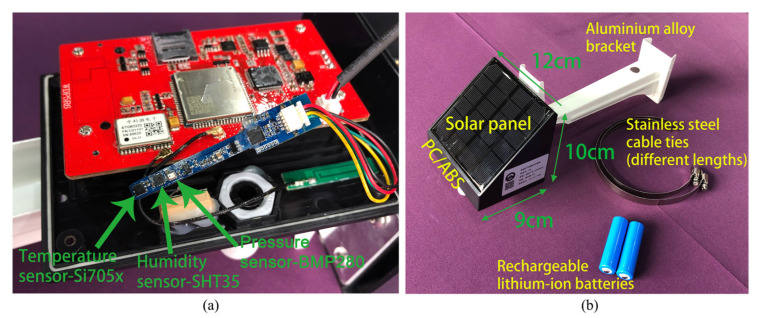
(**a**) Integrated circuit board; (**b**) low-cost integrated sensor.

Located in the middle reaches of the Yangtze River, Wuhan is rich in natural resources and diverse surface types, which makes it a representative and suitable research area. According to public data from the Hubei Provincial Government, Wuhan’s urban population reached 14.77 million by the end of 2021, solidifying its status as a megacity. This study is based on an IoT research initiative utilizing low-cost environmental sensors in Wuhan, a megacity in China. A total of 252 monitoring units were deployed across Wuhan, and these units were designed for cost-effective operation in real-world environments [51]. Some of these sensors were deployed near standard meteorological stations as site scales for comparison and validation, and the sensors used in this study belong to this category. The low-cost sensors used in this study were innovatively integrated and developed by us. As shown in Figure 1, the sensors are compact, durable, and easy to install. They can monitor multiple meteorological elements such as temperature, humidity, and pressure, and have functions such as timing, positioning, and wireless data transmission. The details of the sensor elements used are shown in Table 1. It is worth mentioning that these sensors do not rely on wired power, but are powered by lithium batteries and solar energy, providing sufficient energy independence and saving energy consumption, which is described in more detail in our previous research [51]. The integrated design allows for the real-time wireless transmission of data, with user-adjustable sampling intervals. For this experiment, the low-cost sensors were set to sample data once an hour, which was aligned with the period reported by standard weather stations. Data preprocessing included removing duplicates due to multiple responses and missing values due to instrument failure.

**Figure 2:**
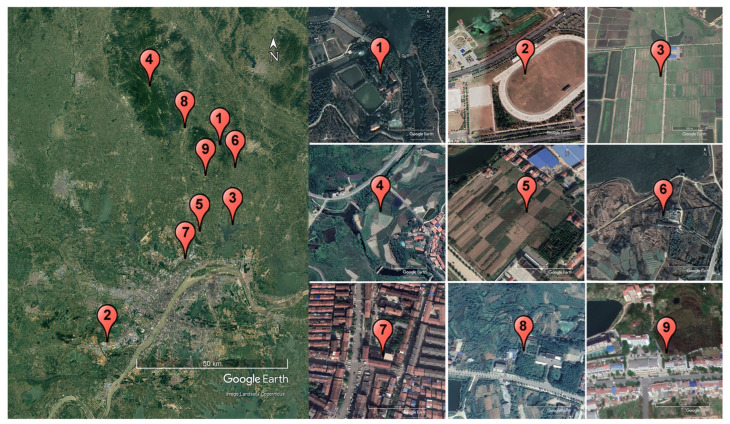
Location map of nine experimental sites in Wuhan, with surrounding scenes of each low-cost sensor deployment.

Sensor deployment adhered to principles of wide spatial distribution, coverage of diverse scenarios, and proximity to standard monitoring stations. The nine experimental sites within Wuhan ranged from 9 to 86 km apart (see Figure 2). The locations selected for the experiment all have standard weather stations built in place. Our low-cost sensors were deployed together withweather stations, and their observation height and the direction of the solar panels are basically consistent with those of the weather stations. The sensors were deployed across five surface types: cultivated land, shrubs, woods, built-up areas, and grasslands. Data collection spanned from December 2021 to November 2022, covering four seasons in the Wuhan area. The largest valid dataset recorded by a single sensor compromised 8419 h of data.

### 2.2. Methods

**Figure 3:**
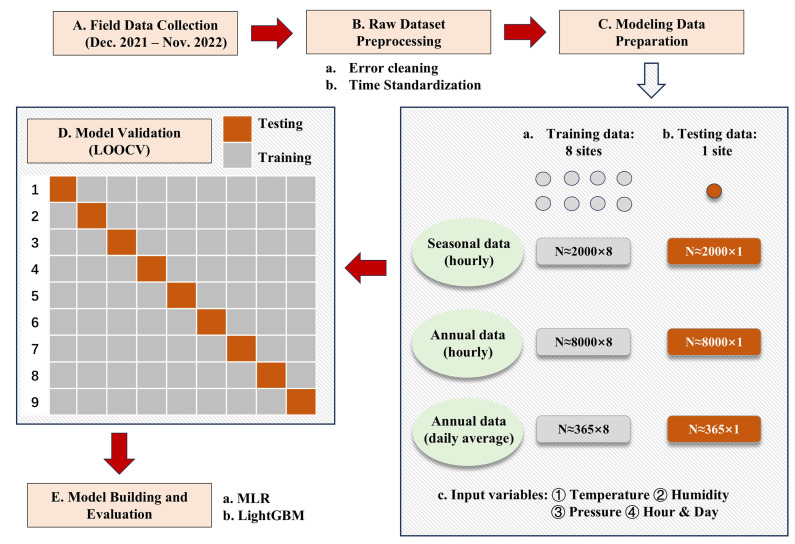
Structure of data processing and calibration.

To evaluate the calibration approach comprehensively, the data were divided into three cases: seasonal data (hourly level), annual data (hourly level), and annual data (daily average level). Seasons were defined according to Chinese regional conventions: spring (March–May), summer (June–August), autumn (September–November), and winter (December–February). The annual data spanned 12 months, from December 2021 to November 2022. Daily average temperatures were calculated using measurements taken at 02:00, 08:00, 14:00, and 20:00. In addition, time was standardized to obtain two feature variables for model input, namely hours and days. The time corresponding to the information collected by the low-cost sensor was extracted, identified as the hours of the day and the days of the year, normalized, and mapped to a 2π period. For seasonal data, the training dataset included approximately 16,000 hourly observations from eight low-cost sensors, while the testing dataset compromised about 2000 observations from another sensor. The annual model’s hourly training dataset contained approximately 64,000 observations, with 8000 being used for testing. The daily average training dataset for the annual model consisted of approximately 2920 observations, and the testing dataset included 365 observations, as shown in Figure 3.

This study employed MLR and LightGBM methods for calibration, with model performance being validated using LOOCV. MLR, a basic yet powerful model, was used to examine the relationships between the explanatory variables and the dependent variable, establishing an additive linear relationship when multiple explanatory variables were involved. However, MLR’s sensitivity to outliers and model instability were noted as potential drawbacks. LightGBM is an adaptive gradient-boosting model that has received widespread attention since it was proposed in 2017 [52]. LightGBM is based on the gradient boosting decision tree (GBDT) algorithm, which uses decision trees as base learners and uses the negative gradient of the loss function as the residual approximation of the current decision tree to fit a new decision tree in the next iteration. Compared with the GBDT method, LightGBM has multiple technical improvements, making it superior in efficiency and accuracy.

Both models were applied to calibrate the raw air temperature data collected by the nine sensors, with the trained models being evaluated using test datasets. The LOOCV approach ensured accurate results by using eight sites as training data and the remaining site as testing data in each iteration, maximizing the use of the available data for validation. In this experiment, nine standard meteorological stations served as reference points, with a low-cost sensor being installed near each station. The air temperature data collected by the standard meteorological stations were used as labels during the model fitting process. To enhance the model’s generalization capability, the training input included only data collected by the low-cost sensors. These inputs compromised temperature, humidity, air pressure, and the previously mentioned time variables, hour and day. This approach ensures that the calibrated model, following comprehensive evaluation, can be effectively applied in areas lacking standard meteorological stations, enabling the efficient calibration of a large number of low-cost sensors.

This experiment employed the LOOCV method for verification. In this approach, with N total samples, N-1 samples are used as training data for each iteration, and the remaining sample serves as the testing data, resulting in N rounds of training and validation. The key advantage of LOOCV is its ability to fully utilize a given dataset, producing results that are both comprehensive and accurate. This method effectively evaluates the model’s generalization ability across different samples while minimizing the impact of randomness, as every sample is used as part of the validation set. This eliminates biases caused by specific data splits and ensures robust performance evaluation. Given the relatively limited sample size in this experiment and the critical importance of accurate calibration for each sensor, LOOCV was deemed highly suitable. Specifically, data from eight stations were used for training in each iteration, while data from the remaining station were used for testing, totalling nine rounds of training and verification.

The spatial generalization capability of the model is crucial in calibrating low-cost sensors, as these are installed in areas that lack standard monitoring stations or where relevant data are unavailable. In this experiment, the locations of the training and testing data differ. By recording the surrounding scenes during sensor deployment in the field, the nine sites were categorized into five typical surface types, as shown in Table 2. This study focused on annual data (hourly level) and selected three surface types, namely cultivated land, shrubland, and woodlands, for comparative analysis. The experiment specified one site as the testing data and evaluated the impact of various scenes and distances by selecting data from several sites for training. The experiment was divided into three groups to assess the influence of the surface types and distances on calibration. First, for sites with similar straight-line distances to the testing site, the influence of different surface types on the calibration accuracy was evaluated. Training data were selected from two sites, one sharing the same surface type as the testing site and the other with a different surface type. Second, for sites with the same surface type, the effect of varying the straight-line distances between the testing and training sites was examined. Due to a limited number of sites with identical surface types, this comparison was only feasible for cultivated land. Third, the experiment considered variations in both the surface type and straight-line distance. One site was designated for testing, while the remaining sites were sorted by distance and used as training data. All calculations were performed in the Python 3.7.7, and all codes were written and debugged in the integrated development environment PyCharm 2020.1 x64 version.

### 2.3. Evaluation Metrics

We developed MLR and LightGBM models to calibrate nine low-cost sensors deployed at different locations and used LOOCV for verification to assess the spatial generalization capability of the models. The experiment considered the influence of multiple scenes and distances to provide a comprehensive evaluation of the calibration methods’ performance across different scenarios. The evaluation metrics used in this study include R^2^, RMSE, and MAE. The R^2^ value was computed using Equation (1).

(1) $$R^{2}={\left(\frac{\sum_{i=1}^{n}\left(Y_{i}-\bar{Y}\right)\left(X_{i}-\bar{X}\right)}{\sqrt{\sum_{i=1}^{n}{\left(Y_{i}-\bar{Y}\right)}^{2}}\sqrt{\sum_{i=1}^{n}{\left(X_{i}-\bar{X}\right)}^{2}}}\right)}^{2}$$

where X represents the value estimated by the model, Y indicates the reference value from the standard weather station, $\bar{X}$ signifies the average of the values estimated by the model, $\bar{Y}$ corresponds to the average of the reference values, and n is the number of samples. The closer the R^2^ value is to 1, the better the model calibration. The RMSE was calculated using Equation (2).

(2) $$\mathrm{RMSE}=\sqrt{\frac{\sum_{i=1}^{n}{\left(Y_{i}-X_{i}\right)}^{2}}{n}}$$

where the closer the RMSE is to 0, the better the model calibration. The MAE was calculated using Equation (3).

(3) $$\mathrm{MAE}=\frac{1}{n}\sum_{i=1}^{n}\left|Y_{i}-X_{i}\right|$$

where the closer the MAE is to 0, the better the calibration of the model. Using the original data monitored by low-cost sensors and the reference data from standard meteorological stations, the accuracy of the low-cost sensor data calibrated by different models is evaluated. The evaluation using these three indicators effectively demonstrated the improvement in the low-cost sensor data after correction with our method.

## 3. Results

### 3.1. Differences Between Sensors and Stations

The statistics of the raw air temperature data collected by the low-cost integrated sensors and standard meteorological stations during the experiment are presented in Table 3. The comparison, conducted at the hourly level from December 2021 to November 2022, shows that the median and average values of the low-cost sensors and the standard meteorological stations are similar, with a small difference in the minimum values. However, the maximum values recorded by the low-cost sensors were notably higher than those of the nearby standard meteorological stations. These statistical results indicate a significant deviation when comparing the hourly air temperature data from the low-cost sensors to the reference data from the standard meteorological stations.

**Figure 4:**
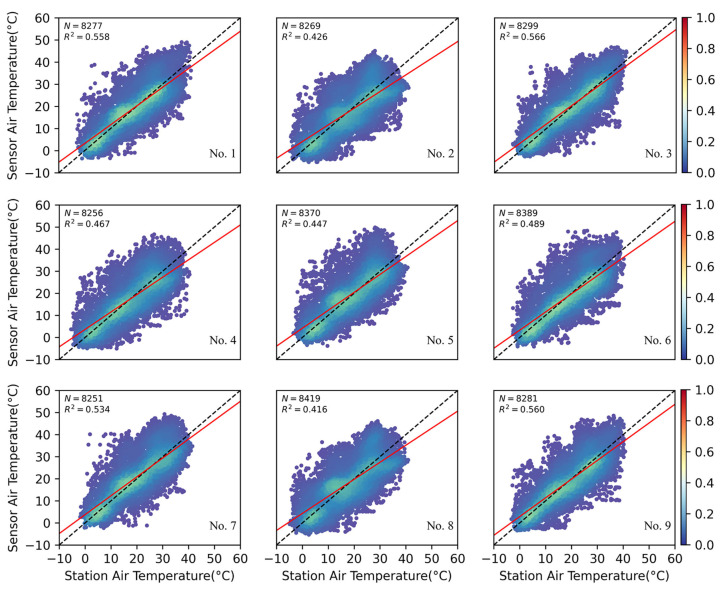
Comparison of air temperature data from nine low-cost sensors with nearby standard weather stations before calibration.

The differences between the low-cost sensors and the standard weather stations at the nine sites are illustrated in Figure 4. We used annual data (hourly level), of which each sensor had more than 8000 samples. The R^2^ value ranged from 0.416 to 0.566. Despite the sensors being located at different sites, their data performance remained relatively consistent. This comparison indicates that, while there are noticeable deviations between the low-cost sensors and the standard weather stations, the long-term trends of the data align closely. Therefore, it is both necessary and feasible to improve the quality of data obtained from low-cost sensors through effective calibration methods.

### 3.2. Calibration and Verification Results

To evaluate the performance of the MLR and LightGBM model, we analyzed the results of calibration at different frequencies (seasonal and annual) and for data types (hourly and daily averages). We also considered the impact of multiple scenes and distances to comprehensively assess the spatial generalization ability of the calibration method.

**Figure 5:**
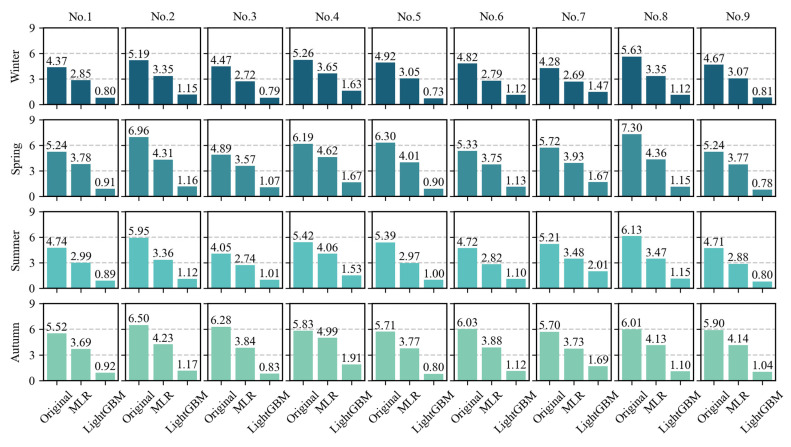
MAE between hourly air temperatures across four seasons, comparing data from the standard weather stations, low-cost sensors (original), MLR-based calibration, and LightGBM-based calibration.

The verification results of the calibration of hourly data from across the four seasons are shown in Figure 5. Each column represents a site, while each row corresponds to a specific season. The histograms in each subfigure represent the original MAE and the MAE results after the MLR and LightGBM calibration. Compared to the original data, both the MLR and LightGBM calibration methods improved the MAE value, with the LightGBM model showing significant advantages. The results also reveal that the original MAE for the stations is better in winter than in the other three seasons. This could be due to the lower air temperature in winter, which reduces the overestimation of high values by low-cost sensors. Overall, the LightGBM method performed well at all sites and in all seasons, confirming the effectiveness of ML-based calibration for hourly seasonal data collected by low-cost sensors at different spatial locations.

**Figure 6:**
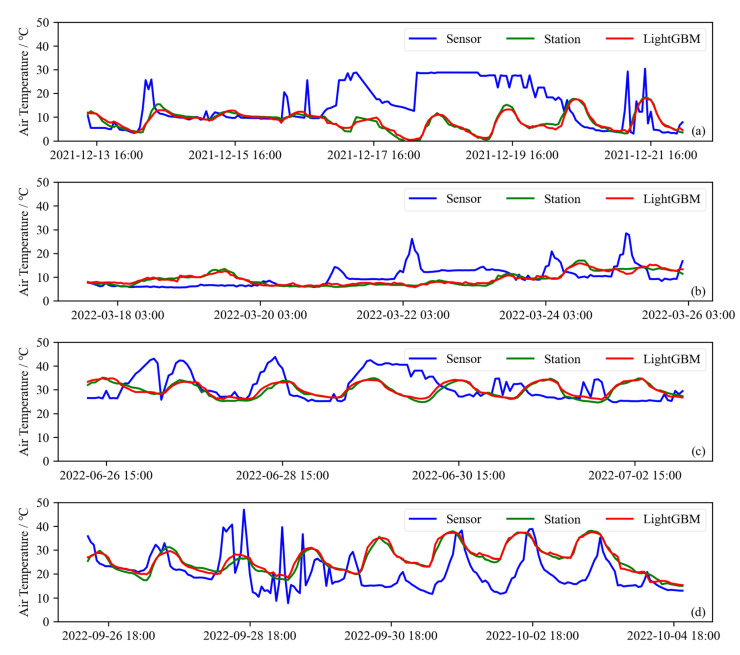
Temporal variation of hourly air temperature data from several consecutive days, randomly selected for low-cost sensors before calibration (Sensor), standard weather stations (Station), and low-cost sensors after LightGBM calibration (LightGBM) across four seasons: (**a**) spring, (**b**) summer, (**c**) autumn, and (**d**) winter. Site No. 5 is used randomly as a demonstration.

To further demonstrate the effectiveness of the LightGBM calibration method, several days were randomly selected from each of the four seasons, and the temporal variation in the data before and after calibration is shown in Figure 6. Hourly air temperature data from consecutive days in four seasons were selected to illustrate how the air temperature data from standard weather stations and low-cost sensors change over time before and after calibration. Site No. 5 was randomly chosen as a demonstration. As can be seen in the middle of Figure 6a, the raw data from the low-cost sensor show abnormal performance, with little variation over time. In this extreme case, the LightGBM calibration method still performs well.

**Figure 7:**
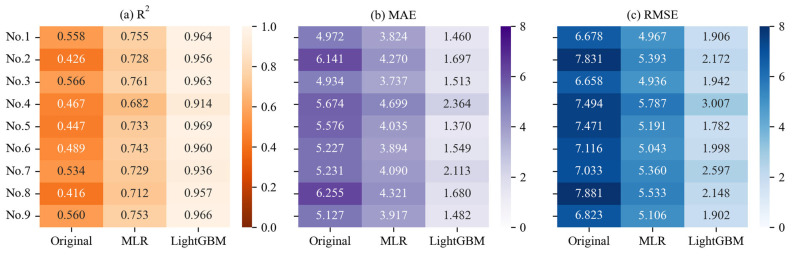
(**a**) R^2^, (**b**) MAE, and (**c**) RMSE values for annual hourly air temperature data from standard weather stations and low-cost sensors (original) after calibration using MLR and LightGBM.

Next, we validated the calibration performance of the MLR and LightGBM models on annual hourly air temperature data. Figure 7 presents the raw and calibrated R^2^, MAE, and RMSE values for the low-cost sensors and standard weather stations at nine sites. After calibration with our LightGBM method, the R^2^ value improved from 0.416–0.566 to 0.914–0.969, the MAE value improved from 4.934–6.255 to 1.370–2.364, and the RMSE value improved from 6.678–7.881 to 1.782–3.007. Notably, the lowest original R^2^ (0.416) was at Site No. 8, which the MLR model improved to 0.712 and the LightGBM model further enhanced to 0.957. These results show that our method offers strong calibration capabilities for hourly temperature data on an annual scale. The LightGBM-based calibration method performed the best, significantly improving the quality of the data from the low-cost sensors and demonstrating clear advantages over the MLR-based calibration method.

**Figure 8:**
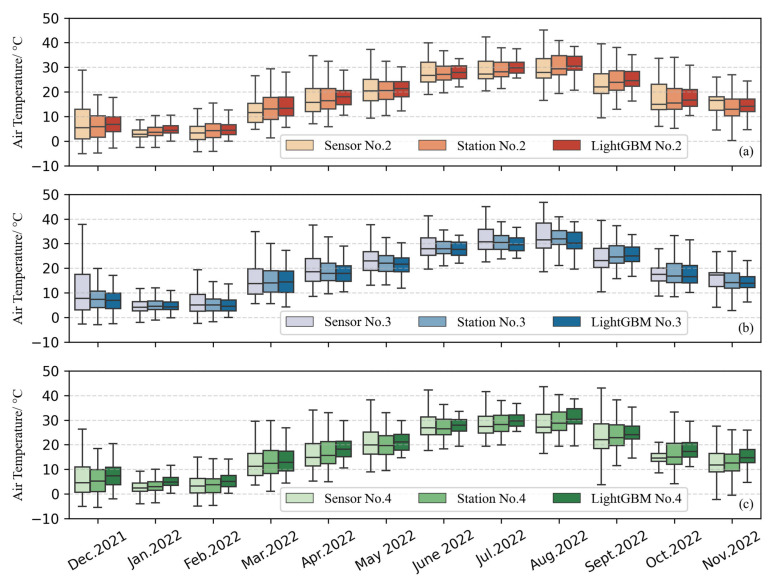
Box plots of hourly air temperature differences between standard weather stations and low-cost sensors across different months. Comparison results of (**a**) No. 2 (located in the south of Figure 2); (**b**) No. 3 (located in the middle and east of Figure 2); (**c**) No. 4 (located in the north of Figure 2).

**Figure 9:**
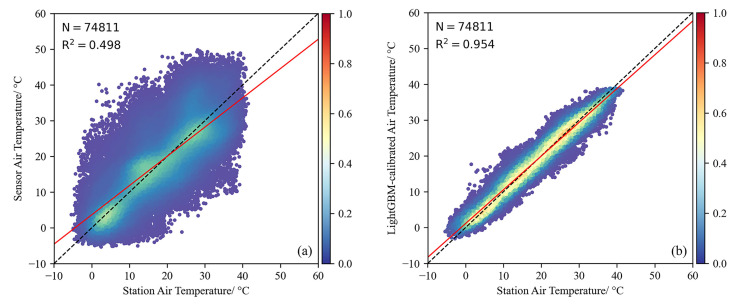
Performance of air temperature data from nine low-cost sensors and standard weather stations (**a**) before and (**b**) after calibration using the LightGBM method.

Although overall evaluation metrics are crucial, the performance of the calibration results over time is also important. Therefore, we present box plots of air temperature (hourly level) by month for low-cost sensors, standard weather stations, and LightGBM calibration in Figure 8. To account for the different spatial locations, we refer to Figure 2, which displays the results for Site No. 2 (southernmost), Site No. 4 (northernmost), and Site No. 3 (central and eastern part). The box plot shows that the quality of low-cost sensor data significantly improves after calibration. Over time, the original errors become more pronounced in the months with higher air temperatures, although the calibration effect was relatively stable. In addition, the comparison results of the annual data (hourly level) of nine sensors at different locations and the data of standard weather stations before calibration are shown in Figure 9a. The model performance after the LightGBM calibration is shown in Figure 9b, where the overall R^2^ can be seen to improve from 0.498 to 0.954, indicating a significant enhancement.

**Figure 10:**
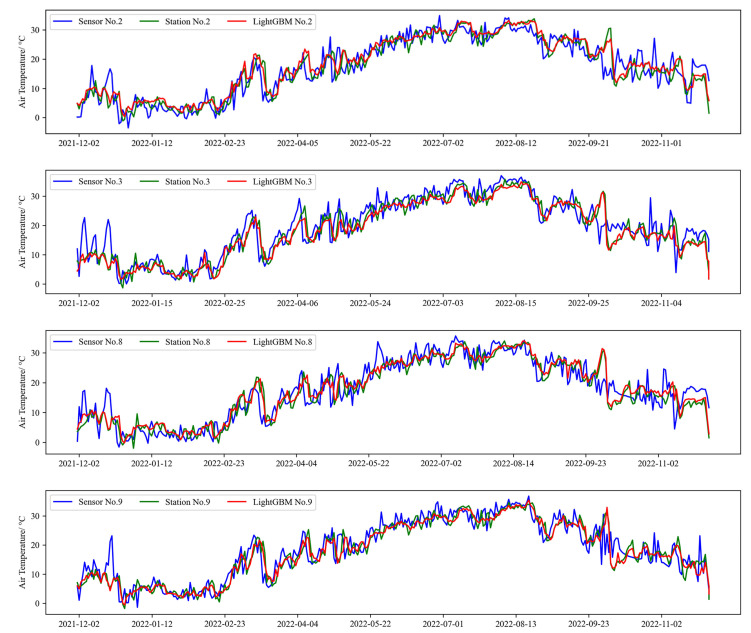
Comparison of raw air temperature data from low-cost sensors (sensor), reference data from standard weather stations (station), and LightGBM-calibrated data (LightGBM) at four randomly selected sites. The long-term daily averages over a 12-month period are displayed.

The effectiveness of the ML calibration method in enhancing the quality of long-term data from low-cost sensors is demonstrated in Figure 10. Four spatially dispersed stations (Nos. 2, 3, 8, and 9) were randomly selected, and their daily average temperatures over 12 months are displayed. Each sub-figure presents the original low-cost sensor data, the reference data from the standard meteorological stations, and the LightGBM-calibrated daily average data. While the trends of the raw low-cost sensor data (blue) are similar to those of the reference data (green), noticeable value discrepancies exist. The LightGBM-calibrated results (red) align closely with the reference data, both in their trends and values. These findings highlight the excellent and stable performance of the ML model in calibrating long-term daily average data.

### 3.3. Differences in Multiple Scenes

To assess the effects of varying scenes and distances on the calibration performance of the tested models, three experiments were designed: (1) evaluating the impact of surface types when the straight-line distances between the training and testing sites are similar; (2) assessing the effect of different distances when the surface types are identical; (3) comparing the results when the surface types differ, with the distances sorted from nearest to farthest.

Firstly, when the testing and training sites have comparable straight-line distances, models trained with data from the same surface type as the testing site demonstrated superior calibration performance. As presented in Table 4, when calibrating sensor No. 5, which was located in cultivated land, two sites with comparable distances from the testing location were selected: Site No. 3, 11 km away, and Site No. 7, 10 km away. Among these sites, Site No. 3, which shares the same surface type as the testing site, yielded better R^2^ results. Similarly, for sensor No. 6, located in woodlands, training data were selected from Site No. 1, 9 km away, and Site No. 9, 10 km away. Site No. 1, which shares the same surface type as the testing site, provided superior calibration outcomes. When calibrating sensor No. 8, located in shrubland, Site No. 9, 17 km away, and Site No. 4, 18 km away, were used as training data. Site No. 9, having the same surface type as the testing site, showed slightly better results.

When the surface types of the training and testing sites are the same, closer straight-line distances result in better calibration performance. For instance, training sites No. 3 and No. 4, located at different distances from testing site No. 5, were used to establish the calibration model. The results in Table 3 indicate that the site closer to the testing site (No. 3) provided a clear advantage in terms of its calibration accuracy.

### 3.4. Performance of High Values

The experimental results indicate that air temperature data recorded by low-cost sensors tend to be overestimated compared to those from standard weather stations, particularly during high-temperature periods. To analyze this further, air temperature data (hourly level) from nine low-cost sensors across various spatial locations were aggregated over 12 months. The observed temperature range for the low-cost sensors was from −5.346 °C to 49.813 °C, so the value range was set to −6–50 °C and divided into seven value intervals, as shown in Table 5. The distribution of the samples within these intervals revealed that the highest temperature interval contained the fewest samples and exhibited poor evaluation metric outcomes. However, it cannot be ignored that the low temperature interval also performed poorly. Consequently, the calibration performance of the LightGBM method was also affected by the quality of the original data, particularly in the more extreme temperature ranges.

## 4. Discussion

In this study, we used LightGBM to calibrate long-term air temperature data collected by low-cost sensors. ML methods have been widely used in the field of low-cost sensor data processing. However, previous research has mainly focused on low-cost particulate matter sensors [36,45], and low-cost meteorological sensors have received comparatively little attention. Nevertheless, the dense meteorological data obtained by these sensors hold immense value for diverse studies, including studies on urban heat islands and crop modeling. These data also play a vital role in urban monitoring and management [25,27], as well as the health and daily lives of urban residents.

Nine sites across Wuhan were utilized as representative locations to calibrate air temperature data from low-cost sensors. To enhance the calibration for long-term data, MLR and LightGBM methods were used, with comparisons being conducted across three data types: seasonal data (hourly level), annual data (hourly level), and annual data (daily average level). The three metrics, R^2^, MAE, and RMSE, revealed that the LightGBM model consistently delivers superior and more stable performance. The LOOCV approach, which allows each sample to serve as a validation set, further underscored the model’s reliability by minimizing the bias associated with specific divisions.

This study also investigated the effects of multiple scenes and distances on the resulting calibration through three experimental scenarios. The results indicated that models which were calibrated using training sites with the same surface type as the testing sites performed better when the distances were similar. However, due to a limited number of sites with similar surface types, there are insufficient experiments on the impact of the distance when the surface types are similar.

Previous studies suggest that closer distances generally improve the verification results, while distant sites may negatively affect the resulting performance. However, calibration experiments often overlook real-world field conditions and variations in distance. This study indicates that distance alone does not have a decisive impact when both the surface type and distance differ. Contrary to the assumption that shorter distances yield better calibration, models applied to more distant sites can still demonstrate superior transmission performance. Therefore, the effective calibration of low-cost sensor models requires a comprehensive evaluation of diverse distances and surface types. Due to the limited availability of sites with uniform surface types, only cultivated land areas were compared, which introduced certain limitations to the findings. Nonetheless, the results remain valuable. When the surface types differ between training and testing sites, they were sorted from near to far according to the straight-line distance to the test site. The calibration effects do not consistently improve with shorter distances, confirming that distance is not a definitive factor in calibration performance when the surface types are different.

Table 4 indicates that the calibration model developed at Site No. 2, located on grasslands, exhibited irregular performance when calibrating three low-cost sensors across different surface types. For instance, despite being the farthest site with a different surface type, Site No. 2 achieved the highest model calibration R^2^ when calibrating site No. 8. Figure 2 reveals that Site No. 2 resembles a playground and is recorded as an artificial horse farm with synthetic turf instead of natural grass according to the actual field deployment records. In contrast to natural lawns, this artificial turf is unaffected by seasonal changes and is significantly affected by horse activities and human intervention. Future research should avoid such unique deployment environments to ensure that site selection is more representative and generalizable. To fully understand the original data and effectively evaluate the calibration model’s performance, no special adjustments were made for low and high values. However, as shown in Table 5, the low- and high-value data will cause a certain degree of adverse effects. Future research should address this issue by increasing the number of sites and samples and incorporating pre-correction methods, such as eliminating or smoothing high-value data, particularly in high-temperature ranges.

The key contributions of this study can be summarized as follows. First, the developed low-cost sensors were tested in multiple real-world, complex field conditions with long-term on-site monitoring. Second, seasonal and annual models were constructed to assess the performance of calibration method over various time scales, including air temperature data at hourly and daily averages. Third, the spatial generalizability of the model was evaluated considering multiple scenarios and distances. Fourth, the model operates independently of standard station data, significantly improving experimental efficiency and enabling calibration in regions without standard monitoring stations. Overall, the LightGBM calibration method exhibits the best performance in improving the quality of data. Our proposed approach is effective and scalable, offering practical value and contributing to the widespread application of low-cost sensors.

## 5. Conclusions

This study proposes and evaluates an efficient calibration method for calibrating air temperature measurements from low-cost sensors under real field conditions. We collected data from multiple spatially distributed sites to assess the method, accounting for variations across time, distance, and environmental conditions. The validation results from seasonal and annual models demonstrate that the proposed LightGBM method significantly enhances the data quality of low-cost sensor data, maintaining excellent and stable performance in datasets of different time scales. Notably, our proposed calibration model is effective even in areas that lack a nearby standard meteorological station, enabling the accurate calibration of low-cost sensors without the need to rely on official data. This contribution highlights the model’s practicality and adaptability in resource-limited regions. This study focuses on identifying the most suitable calibration method for self-developed, integrated low-cost meteorological sensors. It lays the groundwork for further advancements, such as integrating remote-sensing data for near-surface air temperature inversion. In future research, we need to obtain more datasets with similar surface types which are located in different geographical locations and to consider the influence of more environmental factors to improve our methods. Overall, the proposed method is highly significant for calibrating the air temperature measurements made by low-cost sensors in various practical scenarios, improving data quality, and promoting confidence in the application and scalability of low-cost sensors.

## Figures and Tables

**Table 1:** Details of low-cost sensor components.

Type	Manufacturers	Model	Precision
Temperature	Silicon Labs (Austin, TX, USA)	Si705x	±0.1 °C
Humidity	Sensirion (Stäfa, Zurich Canton, Switzerland)	SHT35	±1.5% RH
Pressure	Bosch (Gerlingen, Baden-Württemberg, Germany)	BMP280	±0.12 hPa

**Table 2:** Standard weather station names, low-cost sensor IDs, and surface types for nine sites.

No.	Station	Sensor	Surface Type
1	XJS	005	Woodlands
2	SXY	019	Grasslands
3	LZJ	021	Cultivated land
4	HPYQ	139	Cultivated land
5	HPDT	166	Cultivated land
6	CZJ	175	Woodlands
7	HPSK	249	Built-up areas
8	CXL	283	Shrubland
9	WJH	286	Shrubland

**Table 3:** Statistics and comparison of raw air temperature data from low-cost sensors and standard weather stations. Results are in degrees Celsius.

No.	Type	Min	Max	Median	Mean
1	Sensor	−3.526	49.005	18.021	18.513
	Station	−3.000	41.000	18.200	18.091
2	Sensor	−5.146	45.088	17.531	17.463
	Station	−4.900	40.900	17.800	17.635
3	Sensor	−2.650	46.754	19.047	19.543
	Station	−2.900	40.900	19.000	18.814
4	Sensor	−5.052	46.685	16.658	17.036
	Station	−5.400	40.400	17.150	16.995
5	Sensor	−3.827	49.813	18.364	18.934
	Station	−3.500	40.700	18.300	18.173
6	Sensor	−4.153	48.334	18.510	18.416
	Station	−2.800	40.400	18.600	18.401
7	Sensor	−2.312	49.289	20.045	20.453
	Station	−1.900	41.500	19.700	19.540
8	Sensor	−5.346	46.116	17.357	17.812
	Station	−4.900	40.900	17.500	17.488
9	Sensor	−3.439	48.572	17.685	18.350
	Station	−3.200	40.800	18.500	18.262

**Table 4:** Statistics and comparison of the effects of multiple scenes and distances on calibration performance.

Calibrated	Model	R^2^ (LightGBM)	Surface Type	Distance/km
No. 5 Sensor Cultivated land R^2^ (Original): 0.447	No. 7	0.936	Built-up areas	10
	No. 3	0.956	Cultivated land	11
	No. 9	0.950	Shrubland	18
	No. 6	0.915	Woodlands	24
	No. 1	0.942	Woodlands	29
	No. 8	0.941	Shrubland	35
	No. 2	0.915	Grasslands	48
	No. 4	0.823	Cultivated land	50
No. 6 Sensor Woodlands R^2^ (Original): 0.489	No. 1	0.946	Woodlands	9
	No. 9	0.933	Shrubland	10
	No. 3	0.919	Cultivated land	19
	No. 8	0.925	Shrubland	21
	No. 5	0.896	Cultivated land	24
	No. 7	0.898	Built-up areas	34
	No. 4	0.917	Cultivated land	39
	No. 2	0.979	Grasslands	72
No. 8 Sensor Shrubland R^2^ (Original): 0.416	No. 1	0.935	Woodlands	13
	No. 9	0.937	Shrubland	17
	No. 4	0.917	Cultivated land	18
	No. 6	0.934	Woodlands	21
	No. 5	0.929	Cultivated land	35
	No. 3	0.916	Cultivated land	36
	No. 7	0.888	Built-up areas	43
	No. 2	0.934	Grasslands	75

**Table 5:** The sample size after dividing the air temperature value interval and the index evaluation results before and after calibration.

Value Range/°C	Sample Size	Original			LightGBM-Calibrated		
		R^2^	MAE	RMSE	R^2^	MAE	RMSE
[−6, 2]	4929	−1.246	4.724	6.159	0.785	1.503	1.905
[2, 10]	13,354	0.110	3.884	5.454	0.874	1.569	2.051
[10, 18]	18,578	−0.015	5.134	6.672	0.873	1.819	2.357
[18, 26]	18,029	0.116	5.393	6.926	0.906	1.752	2.257
[26, 34]	13,633	−0.224	5.705	7.656	0.904	1.656	2.147
[34, 42]	5285	−2.389	9.512	11.113	0.877	1.636	2.114
[42, 50]	1003	−6.653	12.691	13.698	0.839	1.530	1.984

## Data Availability

The raw data supporting the conclusions of this article will be made available by the authors on request.
